# Prenatal diagnosis of the recurrent 1q21.1 microdeletions in fetuses with ultrasound anomalies and review of the literature

**DOI:** 10.3389/fgene.2024.1448341

**Published:** 2024-08-29

**Authors:** Lei Liu, Tingying Lei, Fei Guo, Chunling Ma, Li Zhen, Lina Zhang, Dongzhi Li

**Affiliations:** ^1^ Department of Obstetrics, Guangzhou Women and Children’s Medical Center, Guangzhou Medical University, Guangzhou, China; ^2^ Prenatal Diagnostic Center, Guangzhou Women and Children’s Medical Center, Guangzhou Medical University, Guangzhou, China; ^3^ Prenatal Diagnostic Center, Guangzhou Women and Children’s Medical Center, Southern Medical University, Guangzhou, China

**Keywords:** distal 1q21.1 deletions, prenatal diagnosis, chromosomal microarray analysis, ultrasound findings, recurrent 1q21.1 microdeletion syndrome

## Abstract

**Objective:**

The recurrent 1q21.1 microdeletion syndrome is an autosomal dominant disorder and is characterized by dysmorphic facial features, microcephaly, developmental delay, and congenital defects. However, most studies on the distal deletions in the 1q21.1 region were diagnosed postnatally. This study aimed to provide a better understanding of the ultrasound and molecular findings of fetuses with recurrent 1q21.1 microdeletions in prenatal diagnosis.

**Methods:**

In this retrospective study, we reported 21 cases with the recurrent 1q21.1 microdeletion syndrome diagnosed at our prenatal diagnostic center from January 2016 to January 2023. The clinical data were reviewed for these cases, including the maternal demographics, indications for invasive testing, ultrasound findings, CMA results, and pregnancy outcomes.

**Results:**

In the study, a total of 21 cases with recurrent 1q21.1 microdeletions were diagnosed prenatally by CMA. Fifteen cases were described with ultrasound indications, and the most common findings are as follows: increased nuchal translucency (NT) (26.7%), intrauterine growth retardation (IUGR) (26.7%), congenital heart defects (CHD) (20%), and congenital anomalies of the kidney and urinary tract (CAKUT) (13.3%). All the cases with the distal 1q21.1 deletions contain the common minimal region (located between BP3 and BP4) and eight OMIM genes. Parental studies to determine the inheritance of the deletion were performed for eight cases, and half of the cases were inherited from one of the parents. Pregnancy outcomes were available for nine cases; eight (88.9%) pregnancies were determined to be terminated and one (11.1%) was full-term delivery.

**Conclusion:**

To our knowledge, this is the largest study to find that fetuses with recurrent 1q21.1 microdeletions were closely associated with increased NT, CHD, IUGR, and CAKUT. In addition, ours is the first study to report that cerebral ventriculomegaly might be associated with recurrent 1q21.1 microdeletions. More comprehensive studies are needed for a better understanding of the prenatal phenotype–genotype relationship of the recurrent 1q21.1 microdeletion syndrome in future.

## Introduction

The recurrent 1q21.1 microdeletion syndrome (OMIM # 612474) is an autosomal dominant contiguous gene deletion syndrome, which occurs at the 1q21.1 distal region, extending from BP3 to BP4 (GRCh37/hg19: chr1:146533376–147883376), with a size range of 800 kb to 2 Mb, and includes at least eight genes: *PRKAB2*, *FMO5*, *CHD1L*, *BCL9*, *ACP6*, *GJA5*, *GJA8*, and *GPR89B* (Mefford et al., 2008). Individuals with the recurrent 1q21.1 microdeletion have a wide range of clinical manifestations, ranging from unaffected to severely affected. The most common findings include mildly dysmorphic but nonspecific facial features (>75%), mild intellectual disability or learning disabilities (25%), microcephaly (43%), and eye abnormalities (26%) (Edwards et al., 2021; Bourgois et al., 2024). Other findings include congenital heart defects (CHD), congenital anomalies of the kidney and urinary tract (CAKUT), skeletal malformations, joint laxity, seizures (∼23%), and psychiatric conditions such as attention deficit hyperactivity disorder (ADHD), autism spectrum disorder (ASD), and behavioral anomalies (Bernier et al., 2016; Brunetti-Pierri et al., 2008; Digilio et al., 2013; Buse et al., 2017; Upadhyai et al., 2020). However, the cause of the phenotypic variability associated with the deletions remains largely unexplained. Thus, it is challenging to provide genetic counseling for these patients. In addition, most studies on the distal deletions in the 1q21.1 region were diagnosed postnatally, and prenatal reports involving the recurrent 1q21.1 microdeletions were limited (Edwards et al., 2021; Bourgois et al., 2024; Bernier et al., 2016). To better understand these prenatally detected chromosomal microscopic imbalances, we present the findings of ultrasound and molecular analysis of 21 cases with recurrent 1q21.1 microdeletions, especially in pregnant women undergoing prenatal invasive testing, and provide a systematic summary of prenatal phenotypes for such genomic disorders.

## Materials and methods

This study was a retrospective study approved by the Institutional Review Board of the Ethics Committee of Guangzhou Women and Children’s Medical Center, and it met with the ethical standards of experiments on human subjects. Twenty-one prenatal cases of recurrent 1q21.1 microdeletions were recruited in the Prenatal Diagnostic Center, Guangzhou Women and Children’s Medical Center from January 2016 to January 2023. Only the cases with pure distal 1q21.1 deletions were included, and those involving other pathogenic chromosomal microscopic imbalances were excluded. The clinical data were reviewed for these cases, including the maternal demographics, indications for invasive testing, ultrasound findings, chromosomal microarray analysis (CMA) results, and pregnancy outcomes. The data are given as the median (range) or n (%).

The main indications for prenatal diagnosis included ultrasound anomalies, serum screening results for aneuploidy, and advanced maternal age. Fetal samples were collected using amniocentesis (<25 weeks) or cord blood sampling (≥25 weeks or if oligohydramnios was present). Parental blood samples were obtained at the same time. During the study period, with very few exceptions, the CMA was used as a first-tier technology for prenatal diagnosis in fetuses. The CMA platform used was the CytoScan 750 K Array (Affymetrix Inc., Santa Clara, CA, United States), containing 750,436 25-85-mer oligonucleotide probes, including 550,000 nonpolymorphic (NP) probes and 200,436 single-nucleotide polymorphic (SNP) probes (100 Kb resolution). Genomic coordinates are given in GRCh37/hg19. Genomic coordinates in GRCh38/hg38 were remapped to GRCh37/hg19 using the UCSC LiftOver tool (https://genome.ucsc.edu/cgi-bin/hgLiftOver). All patients were offered counseling by a maternal–fetal medicine team, including genetic counselors, before testing and after the diagnosis of 1q21.1 microdeletion.

## Results

**FIGURE 1:**
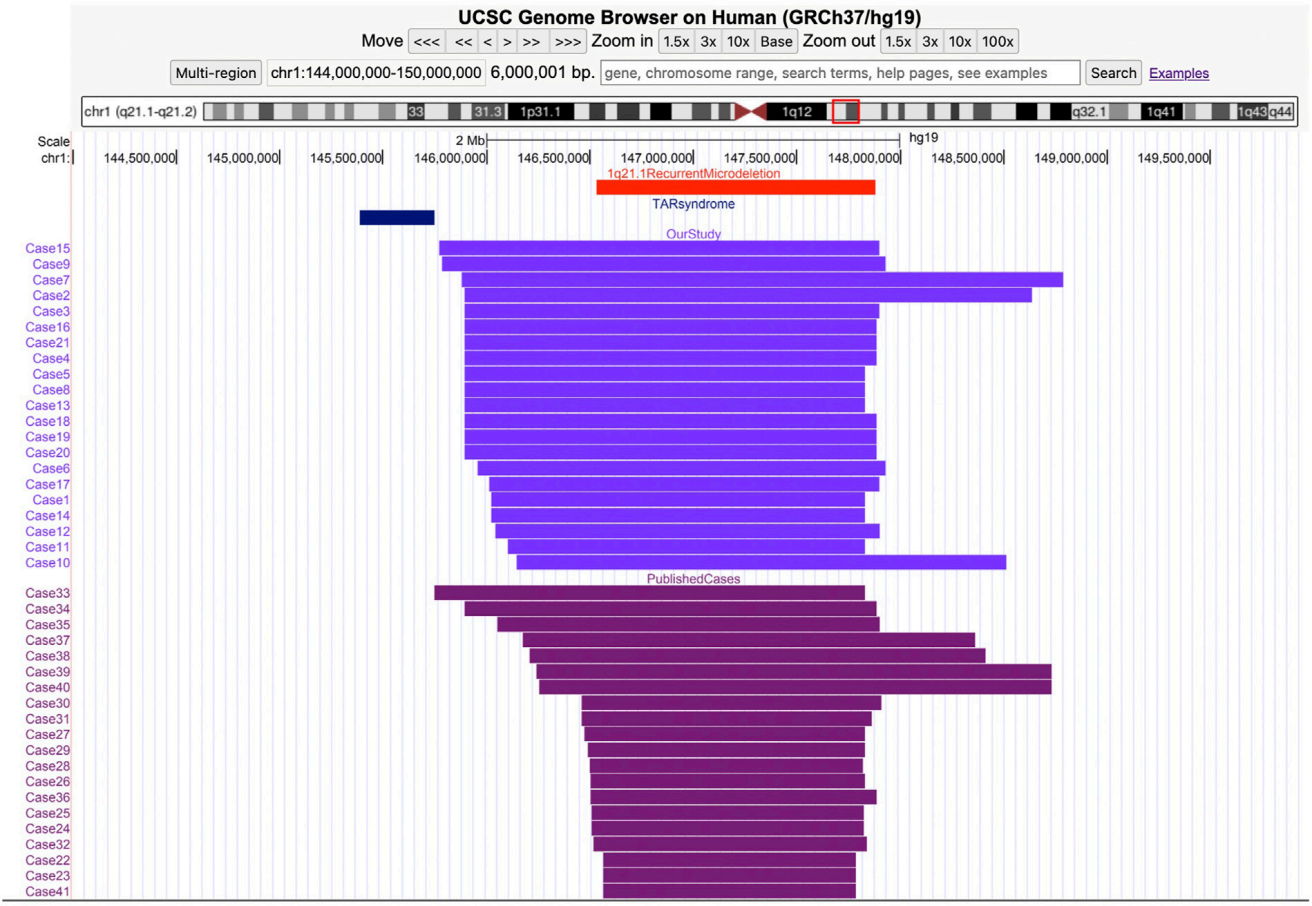
Screenshot from the UCSC Genome Browser (GRCh37/hg19 assembly) showing the coordinates of the 1q21.1 recurrent microdeletion syndrome. Thrombocytopenia absent radius (TAR) syndrome and the 1q21.1 deletions of cases in our study and the previous study.

In the study, a total of 21 cases with recurrent 1q21.1 microdeletions were diagnosed prenatally by CMA (Figure 1). The major fetal and parental clinical indications are summarized in Table 1. The median maternal age was 30 (21–42) years. The median gestational age at prenatal diagnosis was 19 (17–31) weeks. Fifteen cases manifested mild-to-moderate prenatal ultrasound indications at different gestational weeks, including increased nuchal translucency (NT) (cases 8–11, 26.7%), intrauterine growth retardation (IUGR) (cases 12–15, 26.7%), CHD (cases 16–18, 20%), CAKUT (cases 16 and 20, 13.3%), bilateral ventriculomegaly (case 19, 6.7%), and polyhydramnios (case 21, 6.7%). For the other seven cases (cases 1–7), prenatal diagnosis was carried out not based on ultrasound indications but on the advanced maternal age (cases 1–5) and the high risk of results of the serological screening (trisomy 21) (cases 6 and 7). Interestingly, there was no obvious abnormality on ultrasound in any of the seven cases according to the subsequent examinations.

**TABLE 1:** Clinical data of fetuses with recurrent 1q21.1 microdeletions detected by CMA in our study.

NO.	Maternal age (years)	Gestational age (weeks)	Ultrasound finding	Other findings for prenatal diagnosis	CMA result (GRCh37/hg19)	Deleted size (Mb)	Inheritance	Pregnancy outcome
Case 1	38	17+	No abnormality identified	Advanced maternal age	arr1q21.1q21.2 (146,023,922–147,830,830) × 1	1.81	-	-
Case 2	40	17+	No abnormality identified	Advanced maternal age	arr1q21.1q21.2 (145,895,746–148,636,898) × 1	2.74	-	-
Case 3	39	19+	No abnormality identified	Advanced maternal age	arr1q21.1q21.2 (145,895,746–147,898,062) × 1	2.0	-	-
Case 4	42	17+	No abnormality identified	Advanced maternal age	arr1q21.1q21.2 (145,895,747–147,885,600) × 1	1.99	*De novo*	TOP
Case 5	37	18+	No abnormality identified	Advanced maternal age	arr1q21.1q21.2 (145,895,747–147,830,830) × 1	1.94	Pat	-
Case 6	33	18+	No abnormality identified	T21 high risk via serological test	arr1q21.1q21.2 (145,956,384–147,929,323) × 1	1.97	*De novo*	-
Case 7	32	17+	No abnormality identified	T21 high risk via serological test	arr1q21.1q21.2 (145,880,142–148,789,835) × 1	2.91	*De novo*	-
Case 8	32	17+	Increased NT (3.0 mm)	-	arr1q21.1q21.2 (145,895,747–147,830,830) × 1	1.98	-	-
Case 9	30	17+	Increased NT (4.5 mm)	-	arr1q21.1q21.2 (145,786,290–147,929,115) × 1	2.14	-	-
Case 10	26	17+	Increased NT (5.1 mm)	-	arr1q21.1q21.2 (146,145,424–148,513,854) × 1	2.37	-	-
Case 11	28	17+	Increased NT (3.7 mm)	-	arr1q21.1q21.2 (146,106,723–147,830,830) × 1	1.72	-	TOP
Case 12	24	29+	IUGR	-	arr1q21.1q21.2 (146,043,713–147,897,962) × 1	1.85	Mat [exhibiting mild intellectual disability and short stature (148 cm)]	TOP
Case 13	25	30+	IUGR, persistent left superior vena cava	-	arr1q21.1q21.2 (145,895,747–147,830,830) × 1	1.94	-	-
Case 14	24	31+	IUGR	-	arr1q21.1q21.2 (146,023,923–147,830,830) × 1	1.81	-	-
Case 15	36	26+	IUGR	-	arr1q21.1q21.2 (145,770,679–147,897,962) × 1	2.13	-	TOP
Case 16	31	24+	Tetralogy of Fallot and unilateral multi-cystic dysplastic kidney	-	arr1q21.1q21.2 (145,895,746–147,885,600) × 1	1.99	-	TOP
Case 17	21	19+	Dextroaortic arch	-	arr1q21.1q21.2 (146,016,526–147,897,962) × 1	1.88	-	TOP
Case 18	26	26+	Ventricular septal defect	-	arr1q21.1q21.2 (145,895,747–147,885,600) × 1	1.99	*De novo*	TOP
Case 19	30	24+	Bilateral ventriculomegaly (12/15 mm) and single umbilical artery	-	arr1q21.1q21.2 (145,895,747–147,885,600) × 1	1.99	Mat	TOP
Case 20	29	27+	Unilateral hydronephrosis (18 mm) and single umbilical artery	-	arr1q21.1q21.2 (145,895,747–147,885,600) × 1	1.99	Mat	Live birth; normal till now (1.5 years old)
Case 21	30	28+	Polyhydramnios	-	arr1q21.1q21.2 (145,895,746–147,885,600) × 1	1.99	-	-

**FIGURE 2:**
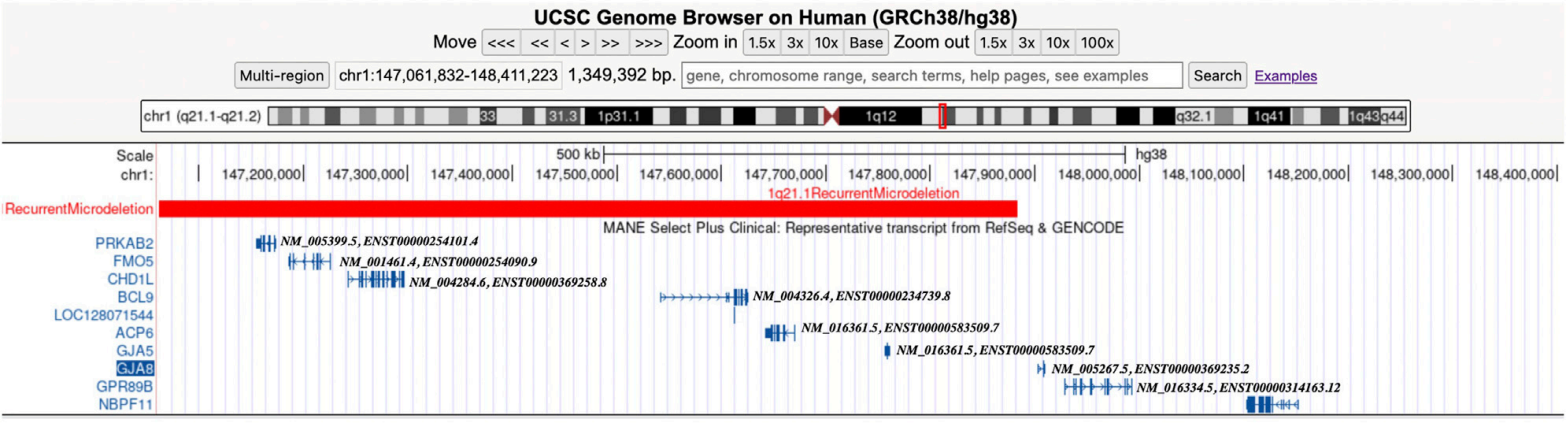
Screenshot from the UCSC Genome Browser (GRCh38/hg38 assembly) showing the location of the 1q21.1 recurrent microdeletion syndrome and the OMIM morbid genes (including MANE Select Plus Clinical transcripts).

In the 21 cases with distal 1q21.1 deletions, the minimum fragment length is 1.72 Mb (Case 11) and the maximum is 2.91 Mb (Case 7). All the segmental deletions contain the common minimal region (1.35 Mb, located between BP3 and BP4) and eight OMIM genes that are unique to the region: *GJA5*, *GJA8*, *ACP6*, *BCL9*, *CHD1L, FMO5*, *GPR89B*, and *PRKAB*2 (Figure 2). Parental studies to determine the inheritance of the deletion were performed for eight cases. Of these, four cases (50%, 4/8) were inherited from one of the parents (cases 5, 12, 19, and 20), and four (50%, 4/8) were confirmed to be *de novo* (cases 4, 6, 7, and 18). The mother of Case 12 exhibited mild intellectual disability and short stature (148 cm), and the parents of seven other cases were asymptomatic. Parental specimens were not available for the remaining 13 cases. Pregnancy outcomes were available for nine cases. Of these, eight (88.9%, 8/9) pregnancies were determined to be terminated; and one (11.1%, 1/9) was full-term delivery, which was a baby girl diagnosed with hydronephrosis and ureteropelvic stenosis. The renal function was normal after surgery, and she is 1.5 years old now and has normal growth.

## Discussion

The recurrent 1q21.1 microdeletion syndrome (OMIM # 612474) is an autosomal dominant disorder usually caused by a recurrent 1.35-Mb deletion in the distal BP3–BP4 region and displays a diversity of clinical phenotypes. In 2008, Brunetti-Pierri et al. (2008) and Mefford et al. (2008) first reported findings of 1q21.1 recurrent deletions in 52 clinical cases, who were associated with certain dysmorphic facial features (such as frontal bossing, deep-set eyes, and bulbous nose), mild-to-moderate intellectual disability, microcephaly, cardiac abnormalities, and cataracts. Since that time, a total of 102 probands with recurrent 1q21.1 microdeletions have been identified by CMA postnatally (Edwards et al., 2021; Bourgois et al., 2024; Bernier et al., 2016). It is estimated that the frequency of the recurrent 1q21.1 microdeletion syndrome is approximately 0.2% of individuals with developmental delays, intellectual disabilities, and/or congenital anomalies (Rosenfeld et al., 2013), following the most commonly reported recurrent deletions: 22q11.2 deletions, proximal 16p11.2 deletions, Williams–Beuren syndrome deletions (7q11.23), and 15q13.3 BP4–BP5 deletions (Dittwald et al., 2013). However, most research studies involving the recurrent 1q21.1 microdeletions focused on postnatal cases, and the prenatal genotype–phenotype association was still unclear due to inadequate reports in the clinic (Yue et al., 2023; Wen et al., 2022). Hence, to provide a better understanding of the deletions in the prenatal setting, we present the ultrasound and molecular findings of 21 cases with recurrent 1q21.1 microdeletions in pregnant women undergoing prenatal invasive testing in the study. To our knowledge, this is the largest study to explore the genotype–phenotype association of the recurrent 1q21.1 microdeletion syndrome in prenatal diagnosis.

The clinical phenotype of the recurrent 1q21.1 microdeletion syndrome is highly variable. It is characterized by dysmorphic facial features, microcephaly, and developmental delay. Several congenital defects, including cardiac, genitourinary, ocular, skeletal anomalies, congenital hypothyroidism, and psychiatric or behavioral abnormalities, have also been described (Buse et al., 2017; Upadhyai et al., 2020). Compared with postnatal phenotypes, prenatal phenotypes involving recurrent 1q21.1 microdeletions were limited in the clinic. To our knowledge, until now, only 20 prenatal cases have been reported (Figure 1) (Bourgois et al., 2024; Yue et al., 2023; Wen et al., 2022; Wang et al., 2017; Chen et al., 2018; Egloff et al., 2018; Su et al., 2022). Prenatal ultrasound findings according to the literature are summarized in Table 2; the common ultrasound features are as follows: increased NT (23.1%, 3/13), CHD (23.1%, 3/13), central nervous system (CNS) abnormalities (23.1%, 3/13), CAKUT (15.4%, 2/13), IUGR (15.4%, 2/13), skeletal anomalies (7.7%, 1/13), and oligohydramnios (7.7%, 1/13). In addition, in the 21 cases, the common ultrasound findings are as follows: increased NT (26.7%, 4/15), IUGR (26.7%, 4/15), CHD (20%, 3/15), CAKUT (13.3%, 2/15), bilateral ventriculomegaly (6.7%, 1/15), and polyhydramnios (6.7%, 1/15). Our findings are mostly consistent with those of prior literature, with the exception that IUGR was more prevalent among our study cohort. Based on the findings mentioned above, we assumed that recurrent 1q21.1 microdeletions were closely associated with fetuses with increased NT (25%, 7/28), CHD (21.4%, 6/28), IUGR (21.4%, 6/28), CAKUT (14.3%, 4/28), and CNS (14.3%, 4/28) in prenatal diagnosis. Additionally, imaging results demonstrate cerebral ventriculomegaly for some 1q21.1 duplication cases, but not deletion cases, in the literature before (Bernier et al., 2016; Yue et al., 2023). In our study, Case 19 was described as having bilateral ventriculomegaly, which will be the first case to be reported with the recurrent 1q21.1 microdeletion, but more evidence should be collected. In summary, our study findings will not only expand the prenatal phenotypes of the deletions but also bring forward the diagnoses of these syndromes and allow couples to make informed decisions about the pregnancy. However, due to the small sample size of the current studies, more efforts should be taken to confirm the prenatal genotype–phenotype association of recurrent 1q21.1 microdeletions in the future.

**TABLE 2:** Clinical data of fetuses with recurrent 1q21.1 microdeletions detected by CMA in the published literature.

NO.	Maternal age (year)	Gestational age (week)	Ultrasound finding	Other findings for prenatal diagnosis	CMA result (GRCh37/hg19)	Deleted size (Mb)	Inheritance	Pregnancy outcome	References
Case 22	26		Encephalomeningocele	-	1q21.1 (146,564,743–147,786,706) × 1	1.22	Mat	TOP	Wang et al. (2017)
Case 23	28		Complete atrioventricular septal defect	-	1q21.1 (146,564,743–147,786,706) × 1	1.22	*De novo*	TOP	Wang et al. (2017)
Case 24	30	22+	Fetal polydactyly of the left foot and echogenic heart foci	-	1q21.1q21.2 (146,507,518–147,824,207) × 1	1.317	Pat	Live birth: a female baby with postaxial polydactyly of the left foot	Chen et al. (2018)
Case 25	33	13+	Increased NT (4.9 mm)	-	1q21.1 (146,506,310–147,824,207) × 1	1.3	*De novo*	Live birth	Egloff et al. (2018)
Case 26	27–34	23–25	Multi-cystic dysplastic kidney	-	1q21.1 (146,501,348–147,828,939) × 1	1.32	Pat	Live birth	Su et al. (2022)
Case 27	27–34	23–25	Ectopic kidney	-	1q21.1 (146,476,526–147,828,939) × 1	1.35	Unknown	TOP	Su et al. (2022)
Case 28	20	17+	No abnormality identified	Following the hint by NIPT	1q21.1q21.2 (146,500,001–1,47,820,000)× 1	1.32	-	Live birth; normal till now (3 months)	Wen et al. (2022)
Case 29	30	18+	No abnormality identified	T21 high risk via serological test	1q21.1q21.2 (146,488,130–147,830,830)× 1	1.34	Pat (microcephaly and normal intelligence)	Live birth; normal till now (10 months)	Wen et al. (2022)
Case 30	30	25+	Ventricular septal defect and racket placenta	-	1q21.1q21.2 (146,460,001–147910000)× 1	1.45	*De novo*	TOP at 28 W	Wen et al. (2022)
Case 31	35	19+	Increased NT (4.5 mm)	-	1q21.1q21.2 (146,460,001–147,860,000)× 1	1.4	*De novo*	TOP at 22 W 3 D	Wen et al. (2022)
Case 32	31	17+	No abnormality identified	T21 high risk via the serological test	1q21.1q21.2 (1,46,520,001–147840000)× 1	1.32	*De novo*	TOP at 21 W 5 D	Wen et al. (2022)
Case 33	27	18+	No abnormality identified	Following the hint via NIPT	1q21.1q21.2 (145,747,846–147,830,830)× 1	2.09	Pat	Follow-up continued	Wen et al. (2022)
Case 34	20	22+	Narrow septum pellucidum and left lateral ventricle dysplasia	-	1q21.1q21.2 (145,895,747–147,885,600)× 1	1.9	*De novo*	TOP at 26 W 5 D	Wen et al. (2022)
Case 35	34	20+	No abnormality identified	Following the hint via NIPT	1q21.1q21.2 (146,053,252–147,898,839)× 1	1.85	*De novo*	Follow-up continued	Wen et al. (2022)
Case 36	20	19+	No abnormality identified	Mother:1q21.1 deletion carrier	1q21.1q21.2 (146,504,399–147,885,600) × 1	1.38	Mat (mother: optic atrophy and retinal detachment Father: difficulty in moving and normal intelligence)	TOP at 23W 1D	Yue et al. (2023)
Case 37	31	16+	IUGR and microcephaly (35 w 6 days)	NIPT infers a high risk of chromosome 16	1q21.1q21.2 (146,174,424–148,358,701) × 1	2.63	Mat	Live birth	Yue et al. (2023)
Case 38	31	18+	Increased NT (3.1 mm)	Abnormal child-bearing history (boy: developmental delay and intellectual disability presenting 46, XY, t (1; 6) (p22; q21) and 1q21.1 microdeletion)	1q21.1q21.2 (146,209,793–148,413,447) × 1	2.2	*De novo*	TOP at 23W	Yue et al. (2023)
Case 39	26	17+	No abnormality identified	NIPT infers a high risk of chromosome 9	1q21.1q21.2 (146,242,158–148,731,429) × 1	2.48	-	Live birth	Yue et al. (2023)
Case 40	28	29+	Aberrant right subclavian artery and ventricular apical thin point	-	1q21.1q21.2 (146,256,254–148,731,429) × 1	2.47		Live birth	Yue et al. (2023)
Case 41	-	-	IUGR and oligohydramnios	-	1q21.1 (146,564,743–147,786,706)× 1	1.3	Pat	Death at Day+4	Bourgois et al. (2024)

In the study, we detected 21 cases with distal 1q21.1 deletions, and there are eight OMIM genes of interest within the following regions: *GJA5*, *GJA8*, *ACP6*, *BCL9*, *CHD1L*, *FMO5*, *GPR89B*, and *PRKAB2*. Haploinsufficiency of one or more of the deleted genes likely contributes to the phenotypes associated with pathogenic variants in these genes. Among them, *GJA5* (OMIM * 121013) encodes gap junction protein, a5, and heterozygous pathogenic variants in *GJA5* is associated with familial atrial fibrillation 11 (OMIM # 614049) and atrial standstill (OMIM # 108770) (Gollob et al., 2006). Several studies also found that heterozygous mutations and deletions in *GJA5* have been identified in patients with structural cardiac defects (especially aortic arch anomalies and tetralogy of Fallot) (Guida et al., 2013; Soemedi et al., 2012; Christiansen et al., 2004) and essential hypertension (Wang et al., 2023). Therefore, we speculated that the fetuses (cases 16–18) with CHD may be associated with heterozygous deletions of *GJA5* in the 1q21.1 region. *BCL9* (OMIM * 602597) encodes B-cell CLL/lymphoma 9 and plays a causal role in the development of B-cell malignancies (Kramps et al., 2002). Li et al. (2011) demonstrated that common pathogenic variants in *BCL9* may be associated with schizophrenia, bipolar disorder, and major depressive disorder in the Chinese Han population. A heterozygous pathogenic variant has also been identified in a patient with a left ventricular outflow tract abnormality (Zaidi et al., 2013). Case 17 presented with dextro-aortic arch, which might also be attributed to the loss of the *BCL9* gene in addition to the *GJA5* gene. *CHD1L* (OMIM * 613039) encodes chromodomain helicase DNA-binding protein 1-like, which is highly expressed in the brain, heart, lung, kidney, and stomach (Ahel et al., 2009). Dou et al. (2017) speculated that *CHD1L* can promote neuronal differentiation in hESCs and play an important role in nervous system development. Case 19 was associated with bilateral ventriculomegaly, which might be correlated with *CHD1L* to some degree, but the reliable correlation between *CHD1L* and CNS abnormalities still needs further investigation. Brockschmidt et al. (2012) also found that *CHD1L* plays a role in kidney development and suggested that *CHD1L* may be a candidate gene for CAKUT. Hwang et al. (2014) identified pathogenic variants in the *CHD1L* gene in patients associated with CAKUT. In our study, cases 16 and 20 were associated with CAKUT, which will further confirm that heterozygous *CHD1L* deletion might be associated with fetal CAKUT. Nevertheless, no genes within the distal 1q21.1 region were found to be associated with IUGR in fetuses.

The recurrent 1q21.1 microdeletions have mostly been described as having incomplete penetrance and variable expressivity (Bernier et al., 2016). They can either occur *de novo* or be inherited from a parent. It was reported that 18%–50% of patients with recurrent 1q21.1 microdeletions were *de novo* and 50%–82% were inherited from their parents (Wang et al., 2017). Like several other recurrent microdeletions (e.g., 16p11.2 and 15q13.3), the 1q21.1 recurrent microdeletions can be inherited from asymptomatic or mildly affected parents (Mefford et al., 2008; Bernier et al., 2016). In our study, parental studies were performed to determine the inheritance of the deletion in eight cases, and four (50%) cases were inherited from one of the parents. Of them, one case (Case 5) was with no abnormality identified prenatally and inherited from the asymptomatic father. Unfortunately, Case 5 was lost to follow-up. The other three cases were associated with abnormal ultrasound findings. Case 12 was associated with IUGR inherited from the mother, who was with mild intellectual disability and of short stature. Case 19 was associated with bilateral ventriculomegaly, which was inherited from the asymptomatic mother. Both pregnancies were chosen to be terminated after genetic counseling. Case 20 was associated with unilateral hydronephrosis, which was inherited from the asymptomatic mother. A baby girl was delivered at term and diagnosed with hydronephrosis and ureteropelvic stenosis. The renal function is normal after surgery, and she is 1.5 years old now and has normal growth. The findings in our study again reconfirmed the ambiguity of disease penetrance, diversity of expressivity, and lack of clear genotype–phenotype correlation, especially in prenatal diagnosis. It will be difficult to offer precise genetic counseling and prognostic assessment for prenatal cases. Hence, multi-center collaboration should be adopted to enlarge the sample size to establish a clearer relationship between recurrent 1q21.1 microdeletions and prenatal–postnatal phenotypes in the future. Furthermore, long-term follow-up should also be guaranteed after birth, including autism, intellectual disability, hearing impairments, attention-deficit hyperactivity disorder, seizures, cardiac disease, and motor difficulties.

## Conclusion

In summary, we described the findings of ultrasound and molecular analysis in 21 fetuses, aiming to investigate the relationship between the recurrent 1q21.1 microdeletion syndrome and prenatal phenotypes. This is the largest study to found that fetuses with recurrent 1q21.1 microdeletions were closely associated with increased NT, CHD, IUGR, and CAKUT. In addition, we are the first to report that cerebral ventriculomegaly might be associated with recurrent 1q21.1 microdeletions. More comprehensive studies are needed for a better understanding of the prenatal phenotype–genotype relationship of the recurrent 1q21.1 microdeletion syndrome in the future.

## Data Availability

The raw data supporting the conclusions of this article will be made available by the authors, without undue reservation.
